# A novel *PITX2* mutation in a Chinese family with Axenfeld-Rieger syndrome

**Published:** 2008-12-05

**Authors:** Dandan Li, Qingguo Zhu, Hui Lin, Nan Zhou, Yanhua Qi

**Affiliations:** 1 Department of Ophthalmology, Harbin Medical University, the Fourth Affiliated Hospital, Harbin, Heilongjiang, China; 2 Harbin Medical University the Second Affiliated Hospital, Harbin, Heilongjiang, China

## Abstract

**Purpose:**

Axenfeld-Rieger syndrome (ARS) is an autosomal dominant disorder characterized by extraocular anomalies and developmental defects of the anterior segment. *PITX2* (paired-like homeodomain transcription factor 2) is considered the major causative gene. In this study, we characterized the molecular defect in *PITX2* in a Chinese family with ARS.

**Methods:**

Two generations of the family with ARS were enrolled in the present study. In addition to ophthalmologic examinations, polymerase chain reaction (PCR) amplification and nucleotide sequencing of all coding exons of *PITX2* were performed. Exon 5 (region 1) was also sequenced in 100 healthy controls unrelated to the family for comparison.

**Results:**

A novel *PITX2* mutation, c.840G>T, was identified in all affected members of the family with ARS that causes an amino acid substitution from tryptophan to cysteine at codon 86.

**Conclusions:**

We found a novel p.W86C mutation in *PITX2* in a Chinese family with ARS. The tryptophan residue at position 86 is strictly conserved in PITX2a proteins from several species and in homeodomain proteins. We suggest that this mutation in *PITX2* is the cause of typical ARS in patients. Our results may be useful for better understanding of the spectrum of *PITX2* mutations and the role of *PITX2* in the development and progression of ARS.

## Introduction

Axenfeld-Rieger syndrome (ARS; OMIM 180500) is a rare multisystem autosomal dominant disorder, which is characterized by complete penetrance but variable expressivity [1]. ARS encompasses several conditions with overlapping phenotypes including Rieger syndrome, Axenfeld anomaly, and Rieger anomaly [2,3]. The classic ocular defects of ARS include iris hypoplasia, iridocorneal adhesion, corectopia, polycoria, posterior embryotoxon, and other less frequent features such as cataracts, retinal detachment, and microcornea [4,5]. Systemic anomalies associated with ARS include facial malformation (telecanthus, maxillary hypoplasia with protruding lower lip), dental abnormalities, and redundant periumbilical skin. Approximately half of the individuals affected with ARS also develop glaucoma [2,3].

The incidence of ARS is estimated to be approximately 1:200,000 births [6]. Mutations in several chromosomal loci have been implicated in ARS including *PITX2* (paired-like homeodomain transcription factor 2; 4q25; OMIM 601542), *FOXC1* (forkhead box C1; 6p25; OMIM 601090), *PAX6* (paired box gene 6; 11p13; OMIM 607108) and *MAF* (v-MAF avian musculoaponeurotic fibrosarcoma oncogene homolog; 16q24; OMIM 177075). A yet to be identified gene at 13q14 (OMIM 601499) may also be found involved in ARS [7-11].

Full-spectrum ARS is caused primarily by mutations in *PITX2* [1]. PITX2 is a member of the bicoid-like homeobox transcription factor family [7]. The homeobox gene family members play fundamental roles in the genetic control of development, particularly in pattern formation and the determination of cell fate [12-14]. PITX2 contains a 60 amino acid homeodomain with a lysine at residue 50 that is characteristic of the bicoid-related proteins [15-17]. Its expression in neural crest cells is necessary for optic stalk and anterior segment structure development [18]. PITX2 is produced as at least four different transcriptional and splicing isoforms, PITX2a, b, c, and d, which have different biological properties [19]. PITX2a, PITX2b, and PITX2c isoforms contain the same homeodomain and COOH-terminus but differ at the NH_2_-terminus. The PITX2d isoform has a truncated, nonfunctional homeodomain [19]. Although the role of PITX2 in the pathogenesis of ARS has yet to be clearly defined, a deficiency in normal PITX2 protein (haploinsufficiency) is suggested to be one of the major molecular mechanisms for the development of ARS [20,21]. Except a few intronic mutations [22,23], most of the mutations detected in human *PITX2* are point mutations in the homeodomain or COOH-terminal domains. A correlation between the severity of ARS phenotypes and the levels of normal PITX2 protein was also noted [24-26]. In the present study, we analyzed the PITX2a isoform for mutations in a family affected by ARS.

ARS is relatively rare in China. The purpose of this study was to describe the clinical and molecular characteristics of a northeastern Chinese family affected by Axenfeld-Rieger syndrome (ARS).

## Methods

### Study participants

**Figure 1:**
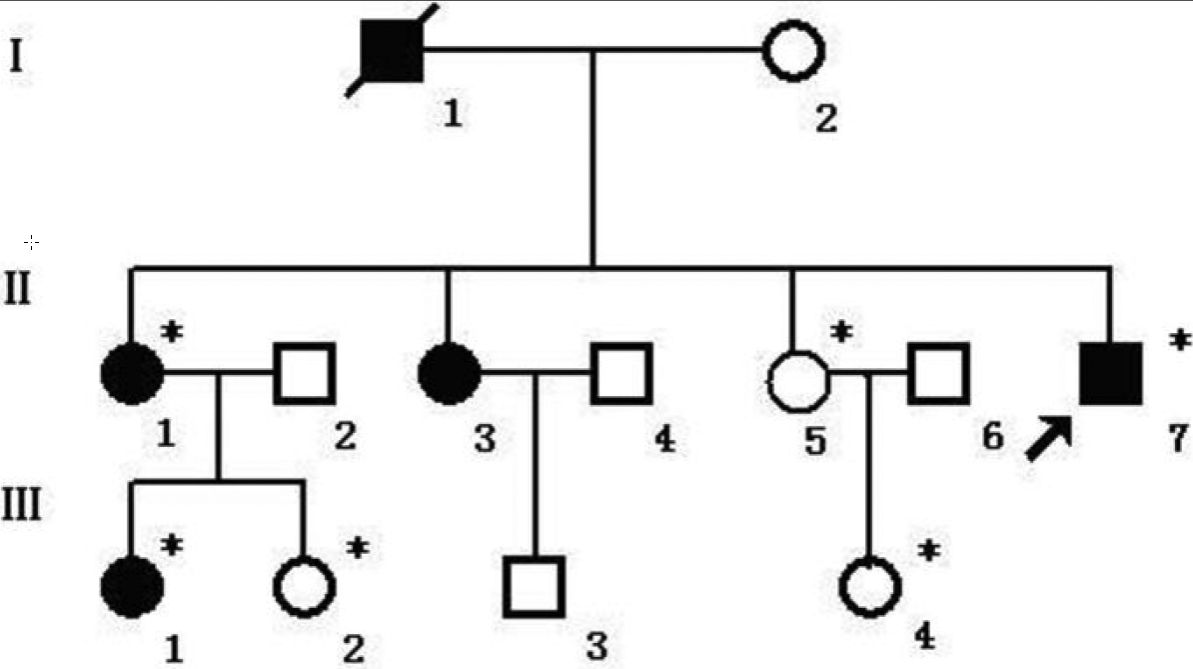
Pedigree of a family with Axenfeld-Rieger syndrome. Autosomal dominant transmission of the disease is evident. The asterisks indicate subjects who underwent clinical and molecular analysis. Black symbols represent affected members. The arrow signals the proband.

We studied two generations of a Chinese family with a history of Axenfeld-Rieger syndrome (ARS) from the Heilongjiang province in northeastern China (Figure 1). Three patients, three non-carrier relatives, and 100 healthy normal controls were recruited for this study. The study was approved by the Institutional Review Board of Harbin Medical University and informed consent was obtained from each of the participants. All subjects underwent full ophthalmologic examination including visual acuity, slit lamp, funduscopy, gonioscopy, and the measurement of intraocular pressure (IOP).

### Genetic analysis

Blood samples were drawn by venipuncture, and genomic DNA was extracted using the TIANamp Blood DNA Kit (Tiangen Biltech Co. Ltd, Beijing, China). All coding exons of *PITX2* were amplified by polymerase chain reaction (PCR) using specific primers (Table 1). The primers for exons 3-5 were adapted from those described by Cella and coworkers [27]. The PCR fragments were purified with a TIANgel Midi Purification Kit (Tiangen Biltech Co. Ltd) and sequenced with an ABI BigDye Terminator Cycle Sequencing kit v3.1, (ABI Applied Biosystems, Foster City, CA).

**Table 1:** Primer sequences and the sizes of their corresponding PCR products.

***PITX2 *exon**	**Sequences (5′-3′)**	**Amplicon (bp)**
*PITX2*-exon 1	F: CCGCTTCTTACAGCCTTCCT	247
	R: CTGGCGATTTGGTTCTGATT	
*PITX2*-exon 2	F: TGGGTCTTTGCTCTTTGTCC	400
	R: GCGGAGTGTCTAAGTTCAAGC	
*PITX2*-exon 3	F: GGGGCAGTAGCCAAGGACT	289
	R: CAGCTAAGCGGGAATGTCTG	
*PITX2*-exon 4	F: GGCATGCTGACGGGAAAG	300
	R: CTGTACCTCCACAACATCCTC	
*PITX2*-exon 5 (region 1)	F: CACTGTGGCATCTGTTTGCT	324
	R: GGACGACATGCTCATGGAC	
*PITX2*-exon 5 (region 2)	F: TATGAACGTCAACCCCCTGT	400
	R: CCATCCGGCAAGGTCCTA	

## Results

### *PITX2* mutation analysis

**Figure 2:**
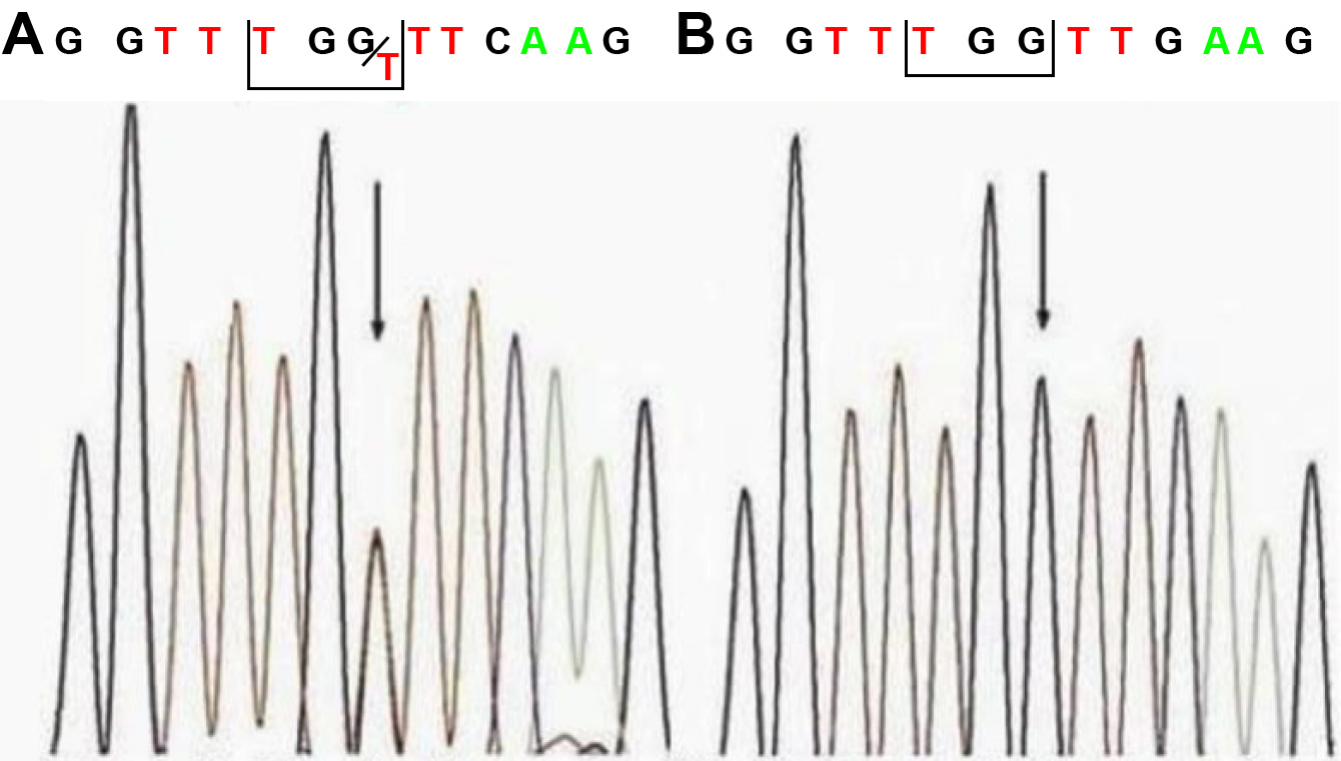
Partial nucleotide sequence of *PITX2*. **A**: The sequence in an affected subject shows a heterozygous G>T transversion (indicated by the arrow). The nucleotide substitution at codon 86 results in a change from tryptophan to cysteine. **B**: Unaffected family members and the general population lack this nucleotide change.

After direct sequencing of *PITX2*, a single heterozygous G to T mutation at nucleotide position 840 of *PITX2* was detected (Figure 2) in all affected members. This resulted in a W86C mutation (tryptophan to cysteine substitution) at the protein level. None of the healthy family members or any of the 100 control subjects was positive for this mutation.

### Clinical findings

**Figure 3:**
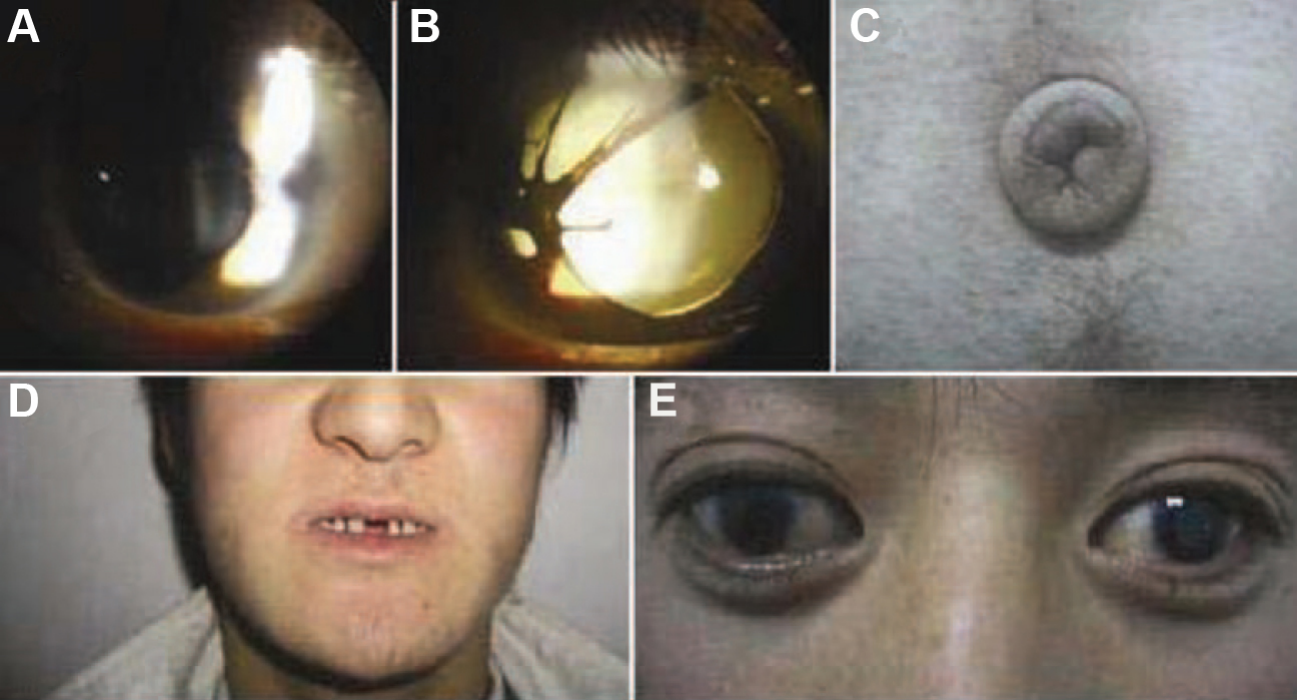
Ocular characteristics and systemic anomalies of the proband with Axenfeld-Rieger syndrome. Slit lamp photographs are shown of the proband showing iris hypoplasia and corectopia associated with partial aniridia in the right eye (**A**), and iris atrophy and polycoria along with congenital cataract in the left eye (**B**). Systemic anomalies of the proband included redundant periumbilical skin (**C**), dental anomalies, a protruding lower lip (**D**), and telecanthus (**E**).

The proband (II:7) was 26 years old. He had experienced defective vision since childhood. His visual acuity was 20/100 in the right eye and counting fingers in the left eye. Although both eyes were affected, the clinical aspects of his eyes were different. In the right eye, ocular examination displayed corectopia associated with partial aniridia and iris hypoplasia (Figure 3A). Funduscopy showed glaucomatous atrophy of the optic nerve, and the IOP was 30 mmHg. External examination of the left eye showed displaced pupils along with iris atrophy, polycoria, and congenital cataract (Figure 3B). The complete cataract prevented ocular fundus examination. The IOP was 26 mmHg. In addition to the various ocular abnormalities, posterior embryotoxon was also found in both eyes following gonioscopic examination. Non-ocular abnormalities of the proband consisted of characteristic facial features with telecanthus, a thin upper lip, and a protruding lower lip (Figures 3D,E). In addition to redundant periumbilical skin, dental anomalies were also present (Figure 3C,D).

Individual II:1, 32 years old, is one of the proband’s sisters. Ocular examination of the right eye showed corectopia and iris hypoplasia. In the left eye, only mild iris hypoplasia was detected. She had posterior embryotoxon in each eye, but the fundus and IOP was normal. The non-ocular abnormalities included dental anomalies and characteristic redundant periumbilical skin. Her daughter, III:1, was nine years old. She had a bilateral posterior embryotoxon unaccompanied by any other ocular abnormality. She had normal teeth but had redundant periumbilical skin. Her IOP was also normal. Individual III:2 who was another daughter of individual II:1, individual II:5 who was another sister of the proband, and her daughter, III:4, did not show any clinical sign of ARS and had normal IOP.

## Discussion

In this study, we described a novel mutation (c.840G>T) in *PITX2a* in a northeastern Chinese family with ARS. None of the proband’s unaffected relatives exhibited the mutation.

PITX2a is a 33 kDa homeodomain protein. The homeodomain is responsible for recognizing specific DNA sequences to bring the transcription factors to proper target genes. This 60 amino acid domain is composed of three helices and a flexible NH_2_-terminal arm [13,28]. Its integrity is essential for binding DNA and is critical for PITX2 to act as a transcription factor. The third helix is also called the recognition helix, which is the site of DNA recognition and binding, and is thought to fit into the major groove of DNA and to establish contact with specific residues of the bicoid binding sequence [13,28-30].

**Figure 4:**
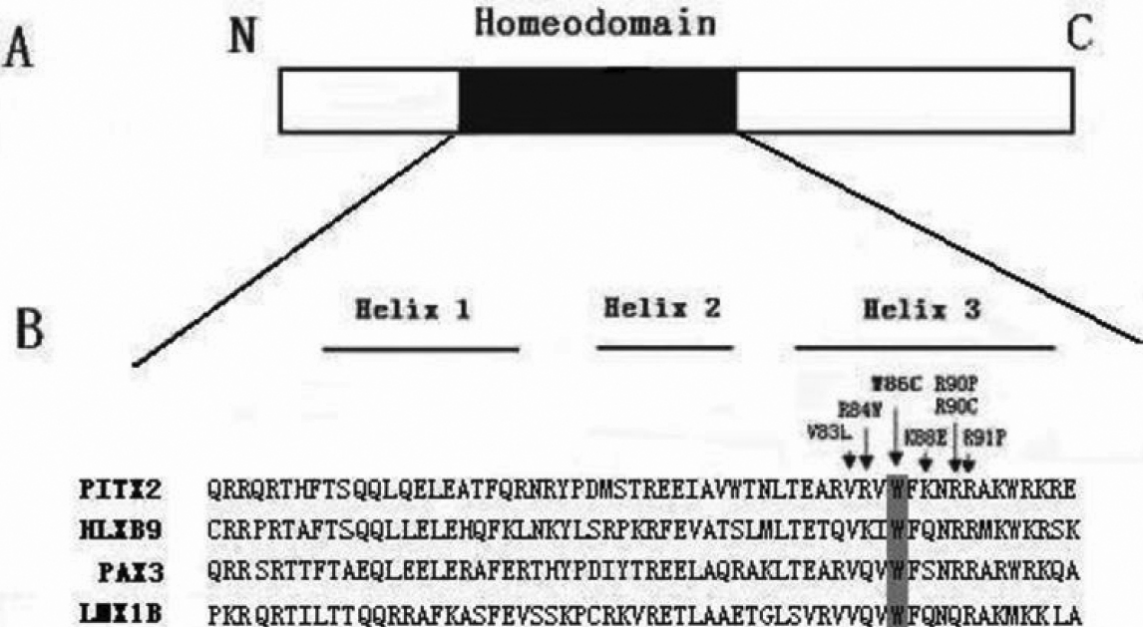
Schematic diagram of PITX2a and amino acid sequence alignments of PITX2 homeodomain with other homeodomain proteins. **A**: The structure of the PITX2a isoform. The black region represents the homeodomain (HD). **B**: Alignments of the human PITX2 homeodomain and related homeodomain-containing proteins (the three α-helices are also indicated) are shown. The arrows indicate the previously characterized mutations within the helix 3 of PITX2a homeodomain. The tryptophan residue at position 86 is conserved among these homeodomain proteins.

The function of the mutated PITX2 proteins can be analyzed in vitro through mutational and functional analyses. It showed that *PITX2* mutations can alter PITX2 nuclear localization, DNA binding, and transactivation activity [31]. Thus far, several missense mutations resulting in single amino acid substitutions within helix 3 of the PITX2a homeodomain have been identified [7,24,32-34] (Figure 4), and these PITX2 mutations alter protein function to varying degrees. For example, the R84W mutant protein has a normal DNA binding specificity and could transactivate the *Dlx2* promoter [25]. However, the K88E mutation is defective in its capacity to bind DNA and transactivation activity [26,35]. In 2002, Quentien et al. [36] demonstrated the R91P PITX2a mutation, which is dominant negative but can still bind DNA. The potential disruption of the helix 3 structure in the R91P mutant may affect the interaction of the factor with a limiting cofactor when bound to DNA. Interestingly, Priston et al. [24] reported the mutation, V83L, in PITX2a affects DNA binding and transactivation differently. Its mutation results in reduced DNA binding but also leads to a greater than 200% increase in transactivation activity compared to wt *PITX2*.

The specific amino acid substitution, a change from tryptophan to cysteine, described in the present paper (p.W86C) occurs in the helix 3 of the homeodomain (Figure 4). The tryptophan residue at position 86 is strictly conserved in PITX2a proteins from different species such as *Xenopus laevis*,* Mus musculus*, *Rattus norvegicus*, *Xenopus tropicalis*, *Canis lupus familiaris*, *Pan Troglodytes*, *Gallus gallus*, and *Bos Taurus* (BLAST). Tryptophan residues are also conserved in other homeodomain proteins such as antennapedia, engrailed, and goosecoid. This high level of conservation indicates that this region may be important for the structure and/or function of the protein. Amino acid changes at this particular residue also have been shown to have a disease-causing effect in the case of the homeodomain transcription factors, HLXB9 (Currarino syndrome), LMX1B (Nail-patella syndrome), and PAX3 (Warrensburg syndrome). The mutational effects may be a disruption of protein stability [37].

In summary, we provide clinical and molecular evidence supporting the occurrence of a novel p.W86C mutation to PITX2a within a northeastern Chinese family with inherited ARS. Our results may improve the understanding of the role that *PITX2* plays in ARS and helps expand the knowledge of the genetic causes of anterior segment disorders.
